# Genome-Wide Screening of Cytogenetic Abnormalities in Multiple Myeloma Patients Using Array-CGH Technique: A Czech Multicenter Experience

**DOI:** 10.1155/2014/209670

**Published:** 2014-06-02

**Authors:** Jan Smetana, Jan Frohlich, Romana Zaoralova, Vladimira Vallova, Henrieta Greslikova, Renata Kupska, Pavel Nemec, Aneta Mikulasova, Martina Almasi, Ludek Pour, Zdenek Adam, Viera Sandecka, Lenka Zahradová, Roman Hajek, Petr Kuglik

**Affiliations:** ^1^Department of Experimental Biology, Faculty of Science, Masaryk University Brno, Bohunice, 62500 Brno, Czech Republic; ^2^Babak Myeloma Group, Department of Pathological Physiology, Faculty of Medicine, Masaryk University Brno, Bohunice, 62500 Brno, Czech Republic; ^3^Department of Internal Hematooncology, University Hospital Brno, Bohunice, 62500 Brno, Czech Republic; ^4^Department of Clinical Hematology, University Hospital Brno, Bohunice, 62500 Brno, Czech Republic; ^5^Faculty of Medicine, University of Ostrava and University Hospital Ostrava, Poruba, 70852 Ostrava, Czech Republic

## Abstract

Characteristic recurrent copy number aberrations (CNAs) play a key role in multiple myeloma (MM) pathogenesis and have important prognostic significance for MM patients. Array-based comparative genomic hybridization (aCGH) provides a powerful tool for genome-wide classification of CNAs and thus should be implemented into MM routine diagnostics. We demonstrate the possibility of effective utilization of oligonucleotide-based aCGH in 91 MM patients. Chromosomal aberrations associated with effect on the prognosis of MM were initially evaluated by I-FISH and were found in 93.4% (85/91). Incidence of hyperdiploidy was 49.5% (45/91); del(13)(q14) was detected in 57.1% (52/91); gain(1)(q21) occurred in 58.2% (53/91); del(17)(p13) was observed in 15.4% (14/91); and t(4;14)(p16;q32) was found in 18.6% (16/86). Genome-wide screening using Agilent 44K aCGH microarrays revealed copy number alterations in 100% (91/91). Most common deletions were found at 13q (58.9%), 1p (39.6%), and 8p (31.1%), whereas gain of whole 1q was the most often duplicated region (50.6%). Furthermore, frequent homozygous deletions of genes playing important role in myeloma biology such as TRAF3, BIRC1/BIRC2, RB1, or CDKN2C were observed. Taken together, we demonstrated the utilization of aCGH technique in clinical diagnostics as powerful tool for identification of unbalanced genomic abnormalities with prognostic significance for MM patients.

## 1. Introduction

Multiple myeloma (MM) is a tumor of postgerminal center isotype switched plasma cells (PCs), which are poorly proliferative but accumulate in the bone marrow leading to anemia, hypercalcemia, and lytic bone disease. Evaluation of genetic lesions associated with prognosis of MM patients is one of the most important diagnostic tools in the field [1]. Detection of chromosomal aberrations by means of standard karyotyping is limited (about 30% of cases) due to resolution and low proliferation activity of PCs [2]. This limitation can be overcome by newer techniques, such as fluorescent* in situ* hybridization (FISH) with detection rate of chromosomal aberrations (CHAs) reaching over 90% of all cases [3]. However, this technique detects only a limited number of specific target sequences and thus provides a very limited view of the genome.

Karyotyping and FISH technique have shown that there are two major genetic subtypes in MM. Hyperdiploid MM (H-MM) is characterized by gains of odd-numbered chromosomes (e.g., chromosomes 3, 5, 7, 9, 11, 15, 19, and 21) and low incidence of* IgH* translocations and it is associated with better prognosis, whereas nonhyperdiploid MM (NH-M) is connected with worse prognosis due to frequent incidence of* IgH* translocations [4]. Several studies described prognostic significance of specific recurrent chromosomal aberrations, such as del(13)(q14)/loss of chromosome 13, del(17)(p13), gain(1)(q21), and* IgH* translocations for MM patients [5, 6]. However, current understanding of MM pathogenesis together with development of modern genome-wide screening techniques proves that common prognostic FISH panels are insufficient for description of genome heterogeneity of malignant PCs [7].

Complete analysis of the MM tumor genome by microarray techniques revealed novel recurrent copy number aberrations, such as deletions in 1p, 6q, 8p, 12q, and 16q, which are now considered additional prognostic factors to high-risk features. In addition, deletions of genes involved in regulation of the NF-*κ*B pathway (*CYLD*,* TRAF3*,* BIRC2*,* and BIRC3*), cell cycle (*CDKN2C*,* CDKN2A*,* and CDKN2B*), or induction of apoptosis (*WWOX and FAF1*) were furthermore described by genome-wide approaches and add important information about genetic changes in MM pathogenesis [8, 9]. Recently, next-generation sequencing (NGS) techniques discovered mutations in several key genes associated with cancerogenesis, such as* K-Ras*,* N-Ras*, or sarcoma viral oncogene homolog B1 (*BRAF*) and postulated a new theory of clonal evolution of MM disease [10].

In our previous studies we showed that incidence of specific cytogenetic abnormalities, such as gain(1)(q21), del(17)(p13), and t(4;14)(p16;q32), detected by FISH in MM patients is connected with shorter overall survival for both newly diagnosed and relapsed MM patients [11, 12]. In this study, we analyzed the genomic profiles of 91 MM samples using oligonucleotide-based aCGH technique. We focused on detailed characterization of CNAs with known prognostic importance as well as CNAs connected with* IgH* translocations and incidence of homozygous deletions in our cohort of patients in terms of better understanding and subclassification of genetic heterogeneity of MM. In addition, we performed validation analysis of the FISH panel routinely used in MM diagnostic [hyperdiploidy, del(13)(q14), del(17)(p13), and gain(1)(q21)] with genome-wide approach to verify the possibility of replacing the standard I-FISH technique due to scalability of genomic profiling.

## 2. Material and Methods

### 2.1. Patients' Characteristics

The bone marrow aspirates from 91 (46 newly diagnosed and 45 relapsed) MM patients were obtained from MM patients between 2007 and 2010 from various centers in Slovakia and the Czech Republic. Patients' clinical features are summarized in Table 1. All patients were included into this study only after they signed the informed consent form approved by the ethical committee of the hospital. The enriched samples of PCs were obtained by either anti-CD138+ immunomagnetic beads (AutoMACS Pro, Miltenyi Biotec GmbH, Bergisch Gladbach, Germany) or by fluorescent-activated cells sorting (FACS Aria, BD Biosciences, San Jose, CA, USA). Detailed protocol of sorting algorithm used in our center was described elsewhere [13]. Briefly, cutoff level of 5% for CD138+ PCs infiltration in the bone marrow was established, and sorting technique (<5% FACS, >5% MACS, resp.) was used according to the manufacturer's instructions.

### 2.2. Microarray Analysis

Genomic DNA (gDNA) for aCGH experiments was extracted using commercially available kit (Puregene Core Kit A, Qiagen) according to manufacturer's protocol. Quality control of gDNA, digestion, labeling, and hybridization steps were performed as previously described [14]. Briefly, 1.0 *μ*g of tumor and reference DNA were independently digested with Alu1 and Rsa1 (Promega, Madison, WI, USA) for 2 hours at 37C. Agilent Euro Female/Male was used as the normal reference in the hybridization experiments. Fluorescent labeling was made by BioPrime Total for Agilent Labeling Module (Invitrogen, Carlsbad, CA, USA) with specific fluorescent dyes Alexa3 for reference and Alexa5 for tumor DNA. Labeled reactions were cleaned up and hybridized at 65C for 24 hours. Human Genome 4×44k CGH Microarrays were scanned by Agilent Surescan C scanner with 5 *μ*m resolution; features were extracted with Feature Extraction software and log⁡⁡2 ratio data were imported and analyzed by Agilent Genomic Workbench 7.0.1.4 (Agilent Technologies, Santa Clara, CA, USA). Aberration calling was made by ADM-2 algorithm [15]. Positive aberration calls were defined by ≥3 consecutive probes and overreaching 0.2-fold change of log_2_ space. We used recommended default threshold 6 for ADM-2 algorithm with accuracy of aberration call confirmed on the basis of known FISH aberrations. The regions with detected CNAs were manually examined due to exclusion of copy number variant (CNV) regions and only copy number changes in exons and microRNA regions were included into further analyses. To identify and eliminate common CNVs from the study, we used default Database of Genomic Variants (http://www.openhelix.com/) for hg18. Physiological loci of the* IgH*,* IgL-k*, and* IgL-l* were also excluded from the analyses. Microarray data are available in the ArrayExpress database (http://www.ebi.ac.uk/arrayexpress/) under accession number E-MTAB-1792.

### 2.3. I-FISH Analysis

The cohort of 91 MM patients was examined for incidence of* IgH* translocations; furthermore, the occurrence of del(13)(q14), del(17)(p13), gain(1)(q21), and hyperdiploidy was compared with aCGH analysis in order to verify the results of whole-genome screening. The detection of PCs in the bone marrow samples was performed by immunofluorescent labeling of cytoplasmic light chain (cIg-FISH), as previously reported [16], or we used CD138+ PCs obtained by cell sorting techniques. The following FISH panel of commercial DNA probes was used for analysis: LSI IGH/FGFR3 dual color probe, LSI 13q14 (RB1) spectrum green probe, LSI p53 (17p13.1) spectrum orange probe, and LSI D5S23/D5S721, CEP 9, and CEP 15 multicolor probe panel (Abbott Laboratories, Abbott Park, IL, USA). Hyperdiploidy was defined as gain of at least two of three evaluated chromosomes in a single cell. Gain(1)(q21) was assessed by homemade probe using fluorescent labeled bacterial artificial chromosome (BAC) (clone RP11-205M9); protocols for BAC isolation and labeling were followed from online resources of University of Bari, Italy (http://www.uniba.it/). Slide preparation and FISH analyses were performed according to manufacturer's protocols. We used cutoff values recommended by the European Myeloma Network [17], 20% cutoff for deletions and numerical aberrations and 10% cutoff for translocations and IgH rearrangements. At least, 100 cells were scored in each sample. Digital image analysis was assessed by fluorescent microscope Olympus BX-61 equipped with a CCD Camera Vosskuhler 1300D and Lucia KARYO/FISH/CGH imaging system (Laboratory Imaging s. r. o., Prague, Czech Republic).

## 3. Results

### 3.1. Summary of Chromosomal Aberrations in 91 MM Patients Detected by I-FISH Technique

The evaluation of unbalanced chromosomal abnormalities (hyperdiploidy, deletion of* RB1*, deletion of* TP53*, and gain/amplification 1q21) and* IgH* translocation t(4;14)(p16;q32) by FISH was performed in 91 MM patients. In our cohort, chromosomal abnormalities were detected in 93.4% (85/91) of cases. Hyperdiploidy was found in 49.5% (45/91); del(13)(q14) was detected in 57.1% (52/91); del(17)(p13) was observed in 15.4% (14/91) and gain(1)(q21) occurred in 58.2% (53/91) of samples. The t(4;14)(p16;q32) was found in 18.6% (16/86); 5 cases were discarded because of low amount of evaluated cells. Incidence of translocation t(4;14)(p16;q32) was associated with nonhyperdiploid (*P* = 0.005) cases as well as gain(1)(q21) and deletion of* RB1* (*P* = 0.025; *P* = 0.052, resp.) and corresponded with simultaneous incidence of del(13)(q14) and gain(1)(q21) (*P* = 0.0047).

### 3.2. Results from Whole-Genome Screening of a Cohort of 91 MM Patients Using Agilent 4×44k Microarrays

**Figure 1:**
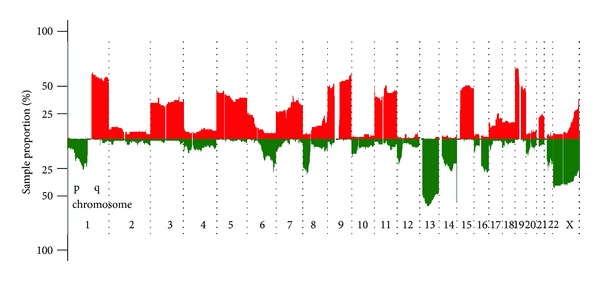
Graphical summary of copy number abnormalities in cohort of 91 multiple myeloma patients. Green color represents areas of loss; red corresponds with areas of gain of genetic material.

The DNA samples from 91 MM patients were analyzed by high-density oligonucleotide aCGH technique. Genome-wide screening using microarrays showed large genomic heterogeneity in MM cases and revealed copy number alterations in 100% (91/91) of samples. Graphical overview of incidence of genomic CNAs is shown in Figure 1. Overall, we found 1557 CNAs (778 gains and 779 areas of loss of genetic material); median was 16 CNAs per patient (range 1–52). The average size of aberration was 26.2 Mbp; 13% (204/1557) of all aberrations were smaller than 1 Mbp. Detailed description of CNAs found in our cohort is available in Supplementary Table 1 (see Supplementary Material available online at http://dx.doi.org/10.1155/2014/209670).

Our results confirm that there are two distinct whole-genome profiles reflecting major genetic subtypes in MM. Incidence of the extra copies of odd-numbered chromosomes is a common feature of hyperdiploid subgroup, which was found in 50.5% (46/91) of samples, whereas 49.5% (45/91) of cases were nonhyperdiploid. Most often duplicated chromosomes were chromosomes 9 and 15 (both 41.8%; 38/91), followed by chromosomes 9 (40.7%; 37/91), 19 (36.3%; 33/91), 5 (33.0%; 30/91), 11 (31.9%; 29/91), 3 (25.3%; 23/91), 7 (22.0%; 20/91), and 21 (18.7%; 17/91).

#### 3.2.1. CNAs with Prognostic Significance in MM Diagnosis Detected by aCGH

*Deletions in 1p*. In our study, we found deletion of 1p in 46.2% of samples (42/91). The whole 1p arm was deleted in 16.7% (7/42) of cases. Furthermore, we identified three frequently deleted areas of 1p.

Most common deleted locus was located in 1p22.1, where we found 480 Kbp minimal deleted region (MDR), which occurred in 32.9% of cases (30/91) and included 5 genes (*HSP90B3P*,* TGFER3*,* BRDT*,* EPHAX4*,* and BTBD8*). The second frequently deleted region was found in 1p32.3 band, where we observed deletion in Fas +associated factor 1 (*FAF1*) and* CDKN2C* (p18) loci in 19.8% of cases (18/91). The third 350 Kbp MDR was detected in 1p12 in 9.9% (9/91), including loci with* MAN1A2*,* FAM46C*, and* GDAP2*.

*Gain 1q*. Regions of gain of genetic material in chromosome 1q were found in 71.4% (65/91) of cases; gain of whole 1q arm was detected in 50.6% (46/91) of patients. In 5.5% (5/91) of samples, we defined 10.9 Mbp minimal region of gain (MRG) in 1q21.2–1q23 including* CKS1B* and* ANP32E*, two genes associated with poor prognosis in MM.

*Chromosome 17*. The deletion in 17p13 locus was found in 14.3% (13/91). We found MDR of 133.5 Kbp covering 4 genes:* ATP1B2*,* TP53*,* WRAP5*, and* EFNB3*. Moreover, we found partial gain in 17q affecting area between 17q21.33 and 17qter in 12.1% (11/91), and in 5 cases (5.5%) we observed incidence of trisomy 17.

#### 3.2.2. CNAs Associated with Chromosomes Involved in IgH Rearrangements

*Chromosome 4*. The loss of genetic material was frequently observed in 4p. The most common MDR was detected in 4p16.3 area in 6.6% (6/91), affecting loci of* FGFR3* and* WHSC1*. Furthermore, we found breakpoint in 4p16.3 locus in 3 cases, resulting probably from unbalanced translocation t(4;14)(p16;q32). In 7.7% of cases (7/91), we defined 7.1 Mbp MDR in 4p15.2, comprising twelve genes (*LGI2*,* SEPSECS*,* PI4K2B*,* ZCCHC4*,* ANAPC4*,* SLC34A2*,* KIAA0746*,* LOC389203*,* RBPJ*,* CCKAR*,* TBC1D19*,* and STIM2).*

*Chromosome 8*. Most common aberration in chromosome 8 was loss of whole 8p, which was found in 23.1% (21/91) of cases. Aberrationsin 8p24.2 affecting* MYC* oncogene were found in 31.9% (29/91) of samples, including both gains and deletions (22%, 20/91; 9.9%, 9/91, resp.). In 7.7% (7/91), we observed breakpoint in the* MYC* locus, resulting most probably from unbalanced t(8;14) translocation.

*Chromosome 11*. We found an extra copy of chromosome 11 in 32.9% of cases (30/91), exclusively in the H-MM group (*P* < 0.001). We identified breakpoint in the* CCND1* locus in 4 cases suggesting incidence of t(11;14). Most often deleted region on chromosome 11 was 11q22 area. We found 4.3 Mbp MDR consisting of 22 genes in 9.9% (9/91) of cases, including loci of two genes with known function in apoptosis, and connected with the NF-*κ*B pathway,* BIRC2* and* BIRC3*, and matrix metallopeptidase cluster.

**Figure 2:**
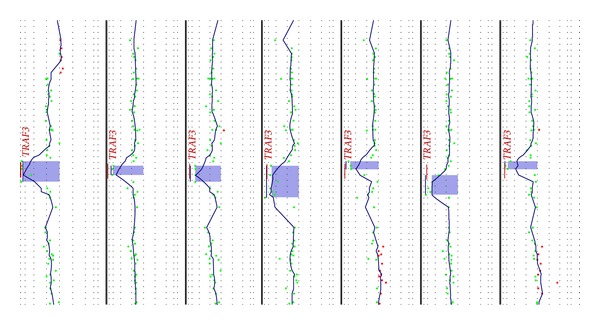
Schematic visualization of homozygous deletions in 14q32.33 region with highlighted* TRAF3* as main target of deletion in this area.

*Chromosome 14*. The most common aberration was monosomy 14, which was observed in 17.6% (16/91) solely in nonhyperdiploid cases (*P* < 0.001). Common MDR was observed in 14q23 region between* AKAP5* and* ADAM21* genes in 8.8% (8/91) of cases. Another MDR was located in 14q32.22 and included 3 genes,* TRAF3*,* AMN*, and* CDC42BPB*. This region was homozygously deleted in incoherent manner (Figure 2) and occurred in 7.7% (7/91) of cases, with size of deletion varying from 48.7 Kbp to 261 Kbp.

*Chromosome 16*. The loss of 16q was the most frequent CNA in our cohort of patients (25.3%, 23/91). In one case with positive t(14;16)(q32;q23), we found interstitial deletion in fragile site FRA16D including* WWOX*. In the short arm of chromosome 16, we found 680 Kbp MDR in 16p13.3 area between* CLUAP1 and NAT15* in 7.7% (7/91).

#### 3.2.3. Other Regions of Recurrent CNAs

*Chromosome 6*. Whereas gains were typical genetic abnormalities for 6p, interstitial deletions were commonly observed in 6q. The most frequently deleted area was 6q25. We defined MDR of size 2.1 Mbp, which was observed in 15.4% (14/91) and was comprised of* OPRM1*,* IPCEF1*,* CNKSR3*,* RBM16*,* TIAM2*,* TFB1M*,* CLDN20*, and* NOX3*. Another region with frequent deletion was 6q16.3, which was found in 11% (10/91) where we defined 3.4 Mbp MDR between* COQR* and* GRIK2*. Partial deletion of 6q (>75%) was also observed in 11.0% (10/91) of cases. In 6p, the most common CNA was gain in 6pter-6p22.3, observed in 8.7% (9/91).

*Chromosome 12*. The most common CNA in chromosome 12 was 69.2 Kbp deletion in 12p13. In 12.3% (13/91) of cases, we found small MDR in 12p13.1 locus including cyclin-dependent kinase inhibitor 1B (*CDKN1B*) and an endothelial cell early response protein gene* APOLD1*. In 5 cases, we observed deletion of whole 12p and 3 cases were missing the whole chromosome 12.

*Chromosome 13*. The loss of genetic material in chromosome 13 was the most common chromosomal aberration observed in our cohort of patients. The monosomy 13 was found in 50.6% (46/91) of cases. In 8.8% (8/91) of cases, we observed 11.2 Mbp MDR between* LRCH1* and* DIAHP3* spanning from 13q14.2 to 13q21.2 and containing genes with important roles in cancerogenesis,* RB1*,* DLEU7*, and miRNA genes* miR-15a/miR-16-1*.

*Chromosome 20*. The most common aberration observed in chromosome 20 was deletion of the short arm. We found loss affecting approximately 2/3 in 20p in 8.8% (8/91) of cases spanning from 20pter to 20p11.23. Gains or losses of whole chromosome 20 were observed in 7.7% (7/91) of samples (4 cases with loss and 3 cases with gain of extra copy of chromosome 20).

*Chromosome 22*. The most frequent CNA in chromosome 22 was loss of the whole chromosome, which was found in 15.4% (14/91) of cases. In addition, 6.6% (6/91) of cases had 33.6 Mbp deletion affecting approximately 1/3 of the 22q arm between centromere and 22q12.2 band.

#### 3.2.4. Regions with Homozygous Deletions Detected by aCGH

The homozygous deletions (HZDs) play an important role in cancer biology and are considered important genetic aberrations as they are able to fully inactivate genes contained within them. In our cohort of patients, HZDs were found in 30.8% (28/91) of cases. Median size was 193 Kbs (range 0.039–1.4 Mbp), and its incidence was noted more often in nonhyperdiploid cases (*P* = 0.02). Incidence of most common HZDs is summarized in Table 2. The most frequently affected region was 14q32.32 with HZD varying from 48.7 Kbp to 261 Kbp observed in 7.7% (7/91) of cases spanning loci of genes (*RCOR1*,* TRAF3*,* AMN*,* and CDC42BPB*) in an incoherent manner. The second most common locus with HZD was 1p32.3 carrying* FAF1* and* CDKN2C*, which was deleted in 5.5% (5/91) of cases. The HZDs in chromosome 13 were also frequently observed. The HZDs were observed in 6.6% (6/91) of cases, but 13q14.2 locus was hit only in 3 cases (3.3%), varying from 52 Kbp to 206 Kbp comprising* RB1, LPAR6*, and* RCBTB2*. Notably, all single case HZDs affected tumor suppressor genes, such as* BRCA2* (13q12.3),* INTS6* (13q14.3), or* SPRY2* (13q31.1). Further loci with occurrence of HZDs were found in a single case in 1p32.3 (*PTPN14*;* ESRRG*), 3q26.3 (*PIK3CA*), 4q22 (*SMARCAD1*), 5q15 (*MCTP1*), 6q22.1 (*KPNA5*), 11q23.2 (*FAM55B*,* CADM1*), 12p31.2 (*CDKN1B*), 16q23 (*WWOX*,* MAF*), and Xq23 (*LHFPL1*,* AMOT*).

### 3.3. Concordance of Unbalanced Chromosomal Aberrations Detected by FISH and aCGH

In order to evaluate the possibility of replacing FISH analyses by aCGH in clinical diagnostics, the occurrence of characteristic recurrent unbalanced chromosomal aberrations was tested. The McNemar test was used for comparison of detection of hyperdiploidy, del(13)(q14), del (17p13), and gain(1)(q21) detected by I-FISH with the incidence of CNAs in those loci from our aCGH aberration list in 91 MM samples.

In our dataset, a total of 36.3% (36/91) of cases were discordant; however, we did not find statistically significant difference between results from both techniques for single aberrations. Detailed overview is shown in Table 3. While in detection of structural aberrations, concordance was over 90%, the most common discrepancy was observed in detection of the ploidy status (14.3%; 13/91). From H-MM cohort evaluated by FISH, 13.3% (6/45) of cases were classified as nonhyperdiploid by aCGH. Similarly, 15.2% (7/46) of NH-MM samples analyzed by FISH were classified as hyperdiploid by aCGH because of the incidence of extra copies of chromosomes undetectable by FISH multicolor panel. Discordant findings were also found in detection of structural aberrations by both techniques. Of the 91 patients with both aCGH and FISH results, aCGH detected 11.0% (10/91) of CNAs that were not detected by FISH [6 cases of del(13)(q14), 2 cases of del(17)(p13), and 2 cases of gain(1)(q21)]. On the contrary, I-FISH detected 11% (10/91) of abnormalities not identified by aCGH [2 cases of del(13)(q14), 3 cases of del(17)(p13), and 5 cases of gain(1)(q21), resp.].

## 4. Discussion

Detection of chromosomal abnormalities is one of the most important independent prognostic markers in MM pathogenesis and prognosis for patients. Similarly to many other types of hematologic malignancies, MM is characterized by numerous structural and numerical genetic lesions involving many oncogenes, tumor suppressor genes or genes involved in signaling pathways important for cell cycle, apoptosis, and so forth. [2]. While karyotyping techniques are able to detect chromosomal abnormalities roughly in 30% of cells because of the low proliferative activity, the introduction of new cytogenetic techniques, such as I-FISH or aCGH, allows us to detect genetic lesions in more than 90% of malignant PCs [18]. Expanded panel of FISH markers includes del(13)(q14)/monosomy 13, t(11;14)(q13;q32), t(14;16)(q32;p23), and hyperdiploidy. Even though I-FISH is nowadays considered as a gold standard for cytogenetic investigations in MM, it may be insufficient for description of given genetic heterogeneity. Moreover, several studies proved virtually 100% occurrence of CNAs in MM when techniques of whole-genome screening were used in MM diagnosis [19]. Based on the above-mentioned studies, we utilized the global assessment of genomic abnormalities via a high-resolution Agilent 4x44k aCGH platform and in combination with FISH in 91 MM patients. To the best of our knowledge, this is the first study of such scale in central Europe in MM patients.

### 4.1. FISH Assessment of Cytogenetic Aberrations Wit Prognostic Significance

Rearrangements of the* IgH* locus play important role in MM pathogenesis. Several studies showed negative prognostic impact of t(4;14)(p16;q32) in newly diagnosed or relapsed patients. In our cohort, we found t(4;14)(p16;q32) using FISH in 17.4% (15/86) of cases in agreement with previous reports [20]. The del(17)(p13) including tumor suppressor gene* TP53* is considered an important negative prognostic factor in MM pathogenesis. In our cohort, we found del(17)(p13) in 15.4% (14/91) of cases in concordance with previous observations [21]. Gain(1)(q21) and subsequently overexpression of* CKS1B* are nowadays considered as an independent prognostic factor in MM diagnosis. In our previous studies, we showed that incidence of this genetic lesion is associated with poor prognosis when detected by I-FISH in both newly and relapsed MM patients. In this cohort, we found gain(1)(q21) in 53% of cases, which is in agreement with previously published results [22].

### 4.2. Whole-Genome Screening by Oligo-Based Microarrays and aCGH Technique

The whole-genome screening using aCGH identified CNAs >100 Kbp in 100% of cases. Most common CNAs were found in 1p, 1q, 6p, 8p, 13q, 14q, 16q, and 22q along with gain of extra copies of odd-numbered chromosomes. Hyperdiploidy was found in nearly half of the cases (47.3%; 43/91). Within the hyperdiploid cohort of patients, we observed incidence of trisomy 11 as well as gain(1q) and del(13q) associated with worse prognosis; however, the association did not meet statistical significance (*P* = 0.112), as previously described [23].

In addition to current high-risk panel genetic abnormalities, several other CNAs associated with adverse prognosis were recently identified by genome-wide techniques. In chromosome 1, deletion in 1p32 affecting loci of* CDKN2C* and* FAF1* is connected with shorter OS. In our cohort, we defined MDR in 1p32.3 locus with incidence of this focal deletion in 19.8% (18/91), which is in good agreement with previous observations [24]. Another frequent deletion in 1p was found in 1p12, including loci of* MAN1A2*,* GDAP2*, and* FAM46C*. Recently, incidence of mutations and deletion of* FAM46C* were described and associated with impaired OS in MM patients [25]. In 1q, we observed common region of gain of genetic sequences in 1q21.2 region with two genes associated with negative impact on prognosis,* CKS1B*, and* ANP32E* [26]. In agreement with previous studies, our results also showed that in most cases, the whole 1q arm is affected [27]. Genetic lesions involving deletions of* TP53* in 17p13.1 were observed in 13.2% (12/91). Even though there is agreement about loss/mutation of* TP53* having negative impact on MM prognosis, MDR in 17p13.1 area in our dataset also included spermine N1-acetyltransferase* SAT2*, which has been reported to be significantly underexpressed in del(17p); it interacts with p65 subunit of the NF-*κ*B pathway and thus is another possible candidate gene in this area [28].

### 4.3. Incidence and Impact of Homozygous Deletions

Homozygous deletions play important role in cancer biology and are considered to be important genetic events. By the definition, this event is able to fully inactivate genes contained within them. The most frequent region associated with incidence of HZD in our dataset was 14q32 (7.7%; 7/91). The incidence of del(14q) is commonly observed in hematological malignancies. In MM, several studies recently showed that TNF receptor-associated factor 3 (*TRAF3*) is an important target of deletion in this locus.* TRAF3* is associated with negative induction of noncanonical NF-*κ*B pathway, enhances* BIRC2*/*BIRC3* mediated proteasome degradation of NF-*κ*B inducing kinase (NIK), and thus increases autonomy of tumor PCs from the bone marrow microenvironment [29]. Similarly to others,* TRAF3* was the target of HZDs in our MDR in 14q32.33; its incidence was comparable with other MM studies as well as other B-lymphomas [30, 31]. Another region affected with HZD was 1q32.3 carrying loci of* FAF1* and* CDKN2C*, which occurred in 5.5% (5/91) of cases. Incidence of HZD in this region is associated with adverse prognosis in MM patients and is also common in mantle cell lymphoma patients [32]. Finally, HZD regions observed in <5% of cases have relevance in MM biology due to involvement in important signaling pathways, such as NF-*κ*B (*CYLD*,* BIRC2*,* and BIRC3*), regulation of cell cycle (*CDKN1B*,* RB1*), or connection with apoptosis (*WWOX*,* FAF1*) [33, 34].

### 4.4. Comparison of FISH Evaluation and aCGH Results in Cohort of 91 MM Patients

Molecular cytogenetic analysis using I-FISH technique is still considered to be a golden standard for cytogenetic evaluation in MM diagnosis. However, genomic profiling using aCGH provides information beyond the commonly detected unbalanced genetic lesions that are observed by FISH. In our cohort, chromosomal aberrations were detected in 93.4% (85/91) of cases using I-FISH, while aCGH screening was able to detect CNAs in 100% (91/91) of cases. The concordance for loss of* RB1 (13q14)*,* TP53 (17p13)*, gain(1)(q21), and hyperdiploidy was 91.2%, 94.5%, 92.3%, and 85.4%, respectively; median of concordance for all aberrations was 91.8%. To our knowledge, no similar study was done in MM, but there are data from different hematological malignancies. Comparative studies in chronic myeloid leukemia (CLL) between FISH and aCGH showed high degree of concordance with our results, with the concordance between FISH and aCGH reaching up to 93% and 95.5%, respectively [35, 36]. Proportion of structural abnormalities missed by FISH and aCGH was 18.7% (17/91) and 17.6% (16/91), respectively, which is a little higher than in previous studies; however, we used a larger cohort of patients and highly purified CD138+ sorted cells as starting material instead of bone marrow samples, which could affect specificity of our analysis over previously published data showing discrepancy from 9 to 12% [37, 38]. In addition, several other studies in MM and CLL also showed that aCGH is less effective when incidence of CNAs is presented to be <30% of the cells [30, 35, 39]. In our dataset, 37.5% (6/16) of cases with missed CNAs by aCGH fell within this condition (2x loss of* RB1*, 2x gain* CKS1B*, and 3x loss of* TP53*). These cases are hard to evaluate by default setting of the analytic software; however, novel computing algorithms developed for detection of mosaic samples are able to overcome this issue [40]. Primary reason for discrepancy in detection of hyperdiploidy was caused by higher false positivity of FISH evaluations, when FISH signals were scored as trisomies when only a part of the chromosome arm was duplicated but not the whole chromosome.

## 5. Conclusions

The results of our study showed that our complex approach comprising cell sorting, I-FISH evaluation of balanced chromosomal changes (*IgH* rearrangement and translocations associated with adverse prognosis for MM patients), and genome-wide profiling gives us a robust diagnostic tool suitable for precise evaluation of the high-risk genetic lesions. The utilization of whole-genome CGH microarrays is able to substitute routine FISH evaluations in detection of unbalanced genetic lesions with prognostic impact in MM and bring additional information about changes in genome of malignant plasma cells even though the detection of clonal aberrations in MM samples could be challenging. Altogether, combination of aCGH and I-FISH technique gives us new opportunities for description of genetic heterogeneity in MM and thus identification of novel cytogenetic features capable of discerning prognosis in MM. However, further studies focusing on genetic background of MM are needed for better understanding and characterization of role of genetic changes in MM pathology.

## Supplementary Material

Description of CNAs which occured in single cases. Table include genomic region (band), start – stop position from given oligonucleotide, size of the lession in Kbp, log2 ratio, and names of genes affected with CNA.

## Figures and Tables

**Table 1:** Patients' baseline characteristics.

Sex	
Males	41
Females	50
Age median	
(at the time of therapy, years); range	69 (38–87)
Follow-up median	
(from therapy, months); range	37.8 (1.7–269.8)
Durie-Salmon stage (from therapy)	
I	9 (9.9%)
II	11 (12.1%)
III	71 (78.0%)
Stages A-B (from therapy)	
A	69 (75.8%)
B	22 (24.2%)
ISS stage (from therapy)	
1	22 (24.2%)
2	25 (27.5%)
3	44 (48.4%)
Ig isotype	
IgG	58 (63.7%)
IgA	15 (16.5%)
IgD	4 (4.4%)
IgM	1 (1.1%)
LC only	13 (14.3%)
Light chains	
Kappa	53 (58.2%)
Lambda	38 (41.8%)
Number of previous treatment lines	
None (first line treatment)	46 (50.6%)
Two	20 (22.0%)
More (>2)	25 (27.4%)
Biochemical parameter (median; min–max)	
Haemoglobin (g/L)	103.50 (66–144)
Thrombocytes (count ×109)	197.50 (27–416)
Calcium (mmol/L)	2.32 (1.47–3.64)
Albumin (g/L)	38.95 (21.1–54.1)
Creatinine (umol/L)	113.00 (54–1136)
*β*2-Microglobulin (mg/L)	5.18 (1.8–42.16)
Lactate dehydrogenase (ukat/L)	3.80 (1.52–22.92)
C-reactive protein (mg/L)	4.20 (0–174)
Plasma cell infiltration of bone marrow (%)	39.4 (0.80–94.60)

**Table 2:** Incidence of most common homozygous deletion in a cohort of 91 multiple myeloma patients detected by array-CGH technique.

Chromosome location	Size (Mb)	Prevalence (%)	Genes
14q32.32	0.063–0.261	7.7	*RCOR1, TRAF3, AMN, CDC42BPB *
1p32.3	0.068–0.387	5.5	*FAF1, CDKN2C *
11q22.1–11q22.3	3.6–4.7	2.8	*BIRC3, BIRC2, MMP cluster *
13q14.2	0.053–0.206	2.8	*RB1, P2RY5, RCBTB2 *
16q12.1-16q12.2	1.40–1.42	1.9	* CYLD, SALL1 *

**Table 3:** Comparison of array-CGH and FISH results in evaluation of cytogenetic aberrations with known effect on prognosis in multiple myeloma patients.

	I-FISH			*P* value	Concordance
aCGH		*del(13)(q14) *			
		Positive	Negative		
	Positive	50	6	*P* = 0.289	**91.2**%
	Negative	2	33		
		*del(17)(p13) *		
		Positive	Negative		
	Positive	11	2	*P* = 1.000	** 94.5**%
	Negative	3	75		
		*gain(1)(q21) *		
		Positive	Negative		
	Positive	48	2	*P* = 0.450	**92.3**%
	Negative	5	36		
		*Hyperdiploidy *		
		Positive	Negative		
	Positive	39	7	*P* = 1.000	**85.4**%
	Negative	6	37		
